# Adenoviral Vector Codifying for TNF as a Co-Adjuvant Therapy against Multi-Drug-Resistant Tuberculosis

**DOI:** 10.3390/microorganisms11122934

**Published:** 2023-12-07

**Authors:** Sujhey Hernández-Bazán, Dulce Mata-Espinosa, Octavio Ramos-Espinosa, Vasti Lozano-Ordaz, Jorge Barrios-Payán, Fernando López-Casillas, Rogelio Hernández-Pando

**Affiliations:** 1 Sección de Patología Experimental, Departamento de Patología, Instituto Nacional de Ciencias Médicas y Nutrición Salvador Zubirán, Mexico City 14080, Mexico; sujheyhb@yahoo.com.mx (S.H.-B.); dulmat@yahoo.com.mx (D.M.-E.); octavioramos13@gmail.com (O.R.-E.); loov1288@hotmail.com (V.L.-O.); qcjbp77@yahoo.com.mx (J.B.-P.); 2 Departamento de Biología Celular, Instituto de Fisiología Celular, Universidad Nacional Autónoma de México, Mexico City 14080, Mexico; fcasilla@ifc.unam.mx

**Keywords:** Tuberculosis, gene therapy, adenoviral vectors, multi-drug resistance, Tumor Necrosis Factor

## Abstract

*Mycobacterium tuberculosis* is the main causal agent of pulmonary tuberculosis (TB); the treatment of this disease is long and involves a mix of at least four different antibiotics that frequently lead to abandonment, favoring the surge of drug-resistant mycobacteria (MDR-TB), whose treatment becomes more aggressive, being longer and more toxic. Thus, the search for novel strategies for treatment that improves time or efficiency is of relevance. In this work, we used a murine model of pulmonary TB produced by the MDR-TB strain to test the efficiency of gene therapy with adenoviral vectors codifying TNF (AdTNF), a pro-inflammatory cytokine that has protective functions in TB by inducing apoptosis, granuloma formation and expression of other Th1-like cytokines. When compared to the control group that received an adenoviral vector that codifies for the green fluorescent protein (AdGFP), a single dose of AdTNF at the chronic active stage of the disease produced total survival, decreasing bacterial load and tissue damage (pneumonia), which correlated with an increase in cells expressing IFN-γ, iNOS and TNF in pneumonic areas and larger granulomas that efficiently contain and eliminate mycobacteria. Second-line antibiotic treatment against MDR-TB plus AdTNF gene therapy reduced bacterial load faster within a week of treatment compared to empty vector plus antibiotics or antibiotics alone, suggesting that AdTNF is a new potential type of treatment against MDR-TB that can shorten second-line chemotherapy but which requires further experimentation in other animal models (non-human primates) that develop a more similar disease to human pulmonary TB.

## 1. Introduction

Tuberculosis (TB) has evolved along with humankind through history, remaining a major health problem; in 2022, there were 10 million new cases around the world [1]. The main cause of TB is *Mycobacterium tuberculosis* (Mtb), which can cause pulmonary infection in the form of asymptomatic latent infection or active progressive disease.

TB treatment is long (6 to 12 months) and involves a combination of antibiotics (first-line) like rifampicin, isoniazid, ethambutol and pyrazinamide; however, potential side effects or toxicity contribute to abandonment and a rise of multi-drug-resistant mycobacteria (MDR-TB). MDR-TB requires a more aggressive treatment with second-line antibiotics (moxifloxacin, ethionamide, amikacin, among others) for 9 to 20 months, which increases the cost and risks of toxicity [2]. Approximately 450,000 new cases of TB resistant to rifampicin were diagnosed in 2021.

Control and elimination of Mtb infection has been associated with Th1 response, so expression of IFN-γ, TNF and IL-12 is crucial during early infection for Mtb containment, especially granuloma formation, by which TNF up-regulation is not only necessary for cytokine expression and cell recruitment, but also for maintenance of the structures that prevent replication, containment and could also lead to Mtb elimination [3].

TNF is a pro-inflammatory cytokine produced mainly by activated macrophages and lymphocytes, but NK cells, eosinophils, endothelial cells and fibroblasts can also be a source of this cytokine. It is involved in several physiological processes like germinal center and immune system development, defense against pathogens and cancer; it is also crucial to granuloma formation [4]. Through TNFR1 and TNFR2 receptors, apoptotic or necrotic pathways can be activated, along with transcription factors like NF-kB and AP-1 promoting expression of pro-inflammatory cytokines (IL-1β and IL-6), chemokines (MCP-1) and inducible nitric oxide synthase (iNOS) [5,6,7]. TNF expression is induced by mycobacteria proteins, such as EsxL, through interaction with TLR2; in turn, TNF expression induces IL-6 production in murine macrophages [8].

Mtb-infected mice treated with anti-TNF antibodies resulted in an increased bacteria load and necrosis, with a deficient granuloma formation when compared to those without any blockade of TNF [9]. This was also observed in humans, where patients with rheumatoid arthritis or Crohn’s disease that received anti-TNF therapy had an increased rate of latent TB reactivation [10,11]. For other autoimmune diseases like ankylosis spondylitis and psoriasis, treatment with TNF inhibitors was associated to an elevated risk for cancer and serious disease infection that includes TB [12]. Mtb induces expression of TNF and IL-10; both cytokines have an opposite role in apoptosis induction, which is a functional process to eliminate mycobacteria; while TNF is a pro-apoptotic molecule, IL-10 is an anti-apoptotic molecule, so the TNF/IL-10 ratio could determine the outcome between apoptosis or macrophage survival during Mtb infection [13].

A sustained and elevated expression of TNF during Mtb infection is crucial to prevent uncontrolled bacterial growth in the lung; considering this, the aim of the present study was to test the therapeutic efficacy of gene therapy with adenoviral vectors codifying for the expression of TNF to potentiate the immune response against progressive infection with MDR-TB; this treatment has already been proved to prevent reactivation in a murine model of latent-like infection [14]. Complete elimination of Mtb in a pulmonary TB progressive model of infection by gene therapy is unlikely, so another aim of the present work was to test a combined therapy with TNF plus second-line antibiotics to seek a faster bacterial clearance, that could shorten the time of therapy, and that could result in less secondary and toxic effects, preventing abandonment of treatment and the surge of drug resistance.

## 2. Material and Methods

### 2.1. Adenoviral Vectors and Determination of Dose

Adenoviral vector construction for TNF protein expression (AdTNF) and control vector codifying only for the green fluorescent protein (AdGFP) have already been described [14,15]. Briefly, a sequence of mTNF was inserted into pAdTrack-CMV (containing GFP sequence as reporter) and co-transformed in *Escherichia coli* BJ5183 with pAdeasy-1 vector. Viral particles were obtained by transfection of HEK-293 cell line (human embryonic kidney cells), purified and titrated by plaque assay as described previously [16].

To determine the dose of adenoviral vector therapy, healthy male 8-week-old BALB/c mice were administered with a low (5 × 10^6^ PFU/mice), medium (5 × 10^7^ PFU/mice) or high (5 × 10^8^ PFU/mice) dose of AdTNF or AdGFP, by intra-tracheal route after sevofluorane anesthesia. After 1, 7 and 21 days of treatment, groups of 3 mice were euthanized by exsanguination under anesthesia with pentobarbital. Lungs were recovered and perfused with absolute ethanol for histological analysis or immediately frozen in 2 mL cryotubes with liquid nitrogen for gene expression quantification.

### 2.2. Experimental Model of Progressive Pulmonary TB and Gene Therapy Administration

The murine model of infection has been described previously [17]. Briefly, male BABL/c mice, 8-week-old were infected with the virulent clinical Mtb strain CIBIN-99, which is an isolate from a patient with advanced pulmonary TB that is resistant to all the first-line antibiotics (MDR-TB). This strain was cultured in Middlebrook 7H9 broth (Difco Laboratories, Detroit, MI, USA). Intra-tracheal administration of 2.5 × 10^5^ bacteria in 100 µL of phosphate buffer saline and 0.02% Tyloxapol with a stainless-steel cannula was performed under anesthesia with sevofluorane. Groups of five mice were maintained in cages fitted with microisolators connected to negative pressure in a P-3 biosecurity-level facility until gene therapy administration.

Disease establishment and progression into a chronic active phase were obtained after 60 days of infection; at this time, gene therapy based on 5 × 10^7^ PFU of AdTNF, AdGFP (control vector) or isotonic saline solution (SS) was administered by intra-tracheal route. After 14, 28 and 60 days, groups of six mice of each condition were selected randomly and euthanized by exsanguination under anesthesia with pentobarbital. Lungs were perfused with absolute ethanol for histologic analysis or immediately frozen in 2 mL cryotubes with liquid nitrogen for bacterial load and gene expression quantification.

For adenoviral therapy combined with second-line antibiotics, 5 × 10^7^ PFU of AdTNF, AdGFP (control vector) or isotonic saline solution (SS) was administered as described above at day 60 post-infection with MDR-TB. The next day (61) after gene therapy, each mouse received daily 0.275 mg of moxifloxacin, 0.1375 mg of ethionamide and 0.4125 mg of pyrazinamide by intragastric route in 100 μL of distilled water, along with 0.4125 mg of amikacin in 10 μL of sterile water by intramuscular route. At days 7, 14, 28 and 60, post-treatment groups of 6 mice randomly selected were euthanized, for each group of treatment, by exsanguination under pentobarbital anesthesia; as indicated previously, lungs were prepared for histologic analysis and bacterial load quantification.

### 2.3. Bacterial Load Quantification in Lungs

Right lungs were frozen immediately after euthanasia by liquid nitrogen immersion. Then, lungs were disrupted using ceramic beads in 2 mL cryotubes, adding 1 mL of PBS-Tween 80 0.05% and homogenized with FastPrepR-24 homogenizer (MP Biomedicals). Serial dilutions of each sample were prepared in a logarithmic order (four in total) and 10 μL was plated on Bacto Middlebrook 7H10 agar (Difco BD, Sparks, MD, USA) enriched with OADC (Difco). Plates were incubated at 37 °C with 5% CO_2_.

Quantification of colony forming units (CFU) was performed at day 21 of incubation. Total bacterial load was calculated considering the dilution factor of two duplicates for each sample.

### 2.4. Quantitative Polymerase Chain Reaction (qPCR)

Gene expression was assessed in left lungs frozen by liquid nitrogen after euthanasia. RNA extraction was performed by disrupting tissue with ceramic beads in FastPrepR-24 homogenizer (MP Biomedicals, Irvine, CA, USA) and RNeasy Mini Kit (Qiagen, Valencia, CA, USA) according to manufacturer instructions. cDNA was synthesized from 100 ng of total RNA with Omniscript Kit (Qiagen).

Real-Time PCR was performed in a 7500 Real-Time PCR System (Applied Biosystems, San Francismo, CA, USA) using QuantiTect SYBR Green Mastermix Kit (Qiagen). Primers sequences for amplification of genes of interest were *Rplp0* (housekeeping gene) F: 5′-CTCTCGCTTTCTGGAGGGTG-3′; R: 5′-ACGCGCTTGTACCCATTGAT-3′, *Tnf* F: 5′-TCGAGTGACAAGCCTGTAGCC-3′; R: 5′-TTGAGATCCATGCCGTTGG-3′, *Nos2* F: 5′-CGCTGGCTACCAGATGCCCG-3′; R: 5′-GCCATAGCGGGGCTTCCAGC-3′, *Ifng* F: 5′-GGTGACATGAAAATCCTGCAG-3′, R: 5′-CCTCAAACTTGGCAATACTCATGA-3′. Cycling conditions were as follows: initial denaturation at 95 °C for 15 min, 40 cycles at 95 °C for 20 s, 60 °C for 20 s and 72 °C for 30 s. The 2^^(−ΔCt)^ method was used for relative quantification of the endogenous housekeeping gene (*Rplp0*).

### 2.5. Histologic Analysis of Pneumonia, Granuloma and Immunohistochemistry

At time points of euthanasia, left lungs of mice were perfused with absolute ethanol and fixed for at least 24 h. Parasagittal sections were dehydrated and embedded in paraffin, in order to obtain sections of 4 µm width. For pneumonia and granuloma observation, hematoxylin and eosin stain were used. Selection and quantification of areas of pneumonia or area of granuloma were performed with an automated image analyzer (Leica Q500/w Image Analysis System; Milton Keynes, UK).

Sections of 4 µm width were deposited on slides covered with poly L-lysine, deparaffinized at 60 °C for 20 min, followed by xylene. Then, slides were hydrated with xylene-alcohol [1:1], absolute ethanol, ethanol 96%, and finally distilled water for 5 min each. Endogenous peroxidase was blocked with 3% peroxide in methanol for 10 min, washed, and Background Sniper (Biocare Medical, Pacheco, CA, USA) was added to block unspecific labeling. Cytokine detection was performed with goat anti-mouse polyclonal antibodies against TNF, IFN-γ and iNOS (Santa Cruz Biotechnology, Santa Cruz, CA, USA), secondary anti-goat antibody-HRP (Goat-on-Rodent HRP-Polymer, Biocare Medical); finally, slides were revealed with diaminobenzidine/H_2_O_2_ and contrasted with hematoxylin stain.

### 2.6. Statistical Analysis

GraphPad Prism 6 Software (GraphPad Software, Inc., La Jolla, CA, USA) was used to analyze data. Two-way ANOVA test was performed for statistical significance between experimental groups at 95% of confidence interval (*p* < 0.05).

### 2.7. Ethical Approval

Animal studies were approved by the Institutional Ethics Committee of Animal Experimentation of the National Institute of Medical Sciences and Nutrition Salvador Zubirán, in accordance with Mexican National Regulations on animal care and experimentation (NOM-062-ZOO-1999).

## 3. Results

### 3.1. AdTNF Dose Determination for Gene Therapy

**Figure 1:**
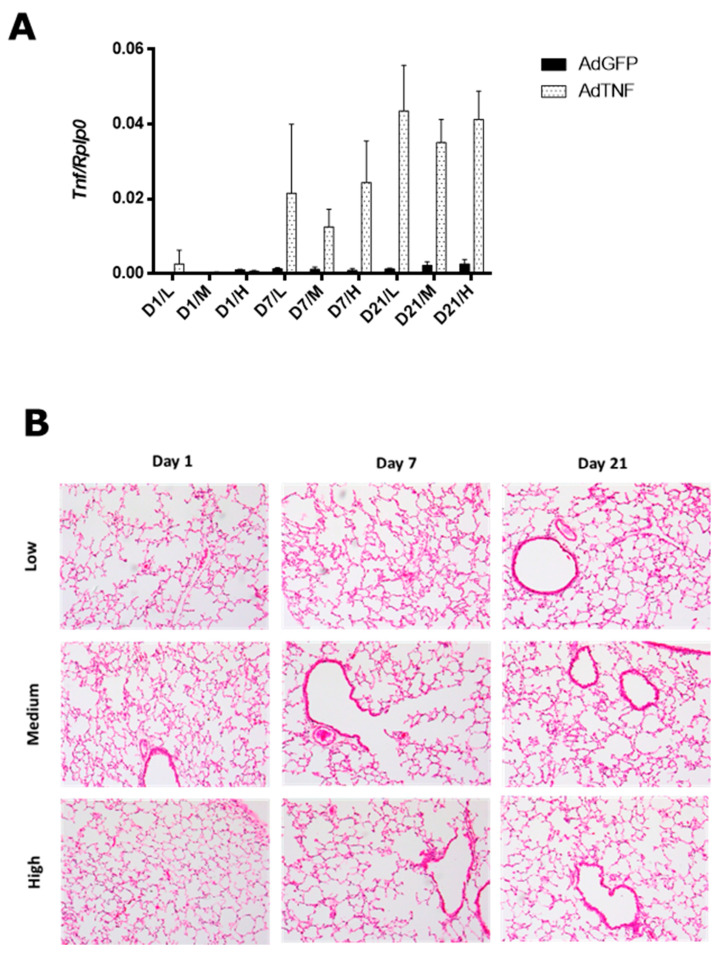
AdTNF dose determination. (**A**) Gene expression of TNF in healthy mice after 1, 7 and 21 days of administration of a low (5 × 10^6^ PFU/mice), medium (5 × 10^7^ PFU/mice) or high (5 × 10^8^ PFU/mice) dose of AdTNF or AdGFP. (**B**) Representative histological features of lungs from mice treated with AdTNF (2.5×).

The adenoviral vector dose was determined by administration of three different doses of AdTNF or AdGFP (low: 5 × 10^6^ PFU/mice, medium: 5 × 10^7^ PFU/mice or high: 5 × 10^8^ PFU/mice) intratracheally, in healthy BALB/c mice. Expression of TNF in the lung by qPCR (Figure 1A) is higher in mice that received AdTNF at day 7 and 21 post-administration, at all doses tested when compared to the group with AdGFP (control vector). However, administration of AdGFP has a minor effect in a dose-dependent manner; this effect was expected since adenoviral vectors are from the 2nd generation, so part of the viral genome is still present, and the expression of some viral proteins can induce a response in mice [18]. At day 7 post-administration, low and high doses had higher expression than the medium dose; nevertheless, mice within each group of the low and high dose had a more variable expression, and a similar pattern is observed at day 21.

Tissue damage in the lung by adenoviral administration was not significant by histological analysis (Figure 1B). At day 7 and 21 post-administration, a slight perivascular inflammation was seen in mice treated with the high dose.

Considering all the results in the histologic analysis and TNF expression, we chose the medium dose (5 × 10^7^ PFU/mice) since there was no lung damage or leukocyte infiltration and the TNF gene expression showed lesser variability than exhibited in the low and high doses.

### 3.2. Therapeutic Effect of AdTNF Administration in a Chronic Phase of a Murine Model of Progressive MDR-TB

**Figure 2:**
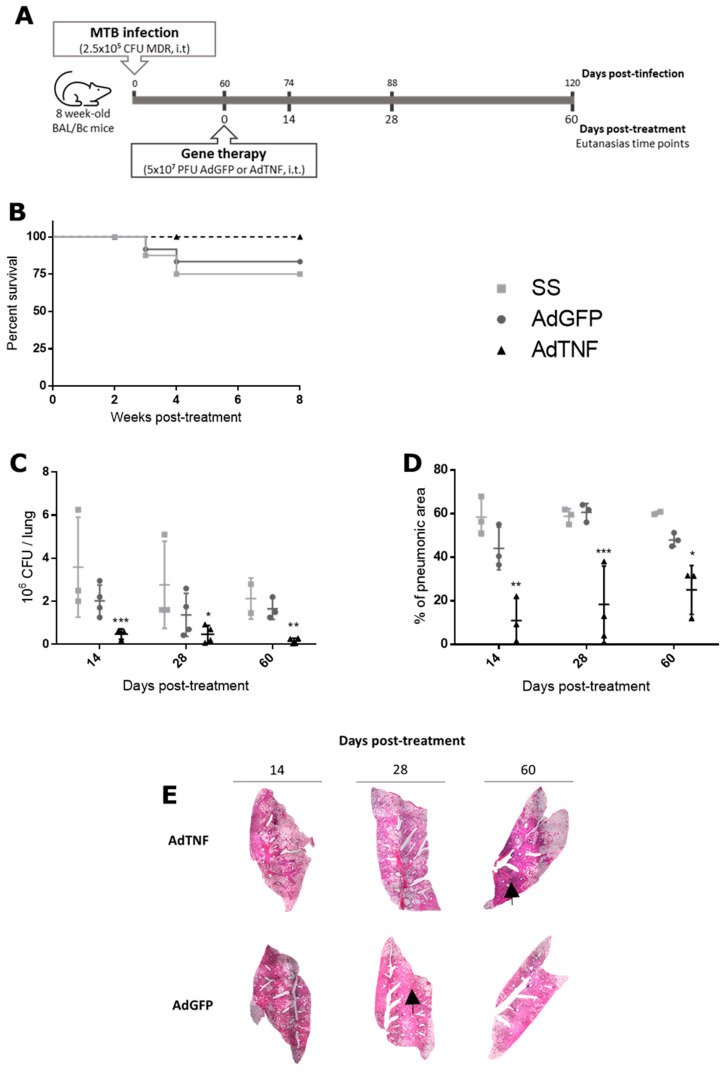
Gene therapy effect of AdTNF in a multi-drug-resistant murine model of infection. (**A**) 8-week-old BALB/c mice were infected intratracheally (i.t.) with 2.5 × 10^5^ CFU of MDR mycobacteria, considering 6 mice per time of euthanasia. A single dose of gene therapy (AdTNF or AdGFP) was administered at day 60 post-infection. Results shown are a representative kinetics from 2 experimental repetitions. (**B**) Survival rates of experimental groups of mice treated with AdTNF, AdGFP or without treatment (SS). (**C**) Bacterial loads in lung determined by colony forming units (CFU). (**D**) Pneumonic area determined by automated morphometry. Asterisks represent statistical significance (* *p* < 0.05, ** *p* < 0.01, *** *p* < 0.001, two-way ANOVA). (**E**) Representative low power micrographs (2.5×) of the lungs of tuberculosis mice treated with AdTNF, AdGFP or saline solution, showing areas of lung consolidation or pneumonia (black arrow).

BALB/c mice were infected with 2.5 × 10^5^ MDR-TB bacteria, then at day 60 post-infection, mice were treated with a single dose of 5 × 10^7^ PFU/mice of AdTNF or AdGFP intratracheally, along with the infection control group that received the vehicle saline solution (SS) (Figure 2A). As shown in Figure 2B, AdTNF therapy allowed for total survival of mice, in contrast to the AdGFP and SS groups, which showed 83% and 75% survival, respectively. In accordance with survival rate, bacterial load (Figure 2C) was lower in the AdTNF group by four times at day 7, three times at day 28 and 10 times at day 60 when compared to the control group (AdGFP).

Pulmonary TB caused tissue damage (progressive pneumonia) that was evaluated by automated histomorphometry. Percentage of pneumonia (Figure 2D) was lower at any time point in mice treated with AdTNF when compared to AdGFP or SS; an inverse effect is observed with respect to time of treatment, being 4, 3.3 and 1.9 times lesser than AdGFP at days 7, 28 and 60 post-treatment, respectively. In Figure 2E, lung consolidation by pneumonia is diminished when AdTNF treatment is given, in contrast to AdGFP (control group).

**Figure 3:**
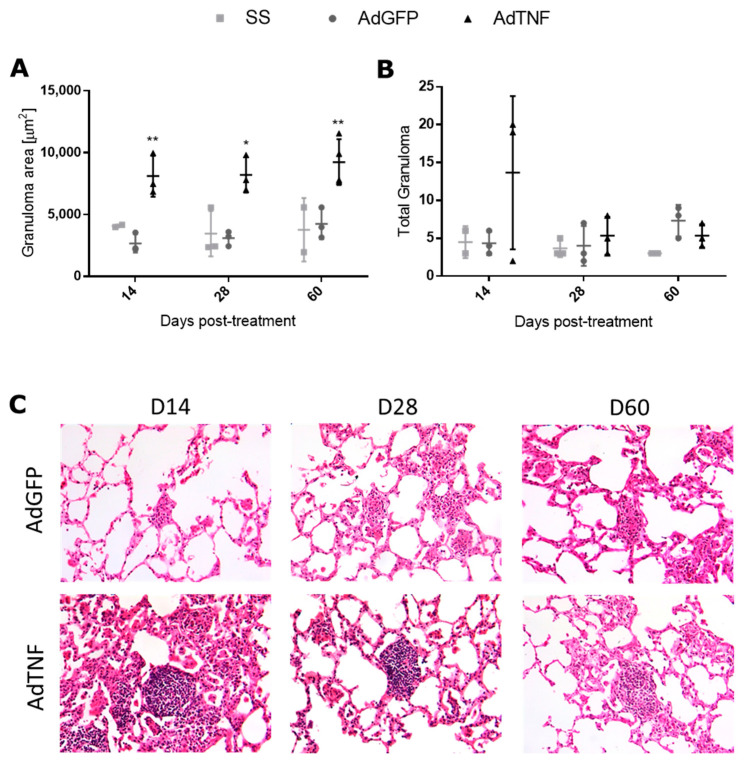
Effect of AdTNF therapy on granuloma formation. (**A**) Granuloma area and (**B**) number of granulomas in lungs of mice treated with AdTNF, AdGFP or saline solution. Asterisks represent statistical significance (* *p* < 0.05, ** *p* < 0.01, two-way ANOVA). (**C**) Representative images (20×) of granuloma of AdTNF or AdGFP; granulomas are larger and more well-preserved in animal treated with AdTNF.

Granuloma formation is important for the containment of Mtb and TNF is a key cytokine in the formation and maintenance of these structures [19]. As observed in Figure 3A, the granuloma area was bigger in mice treated with AdTNF at the three time points analyzed, showing a better formation and mature granulomas that can efficiently contain the bacteria, and was correlated with lesser CFUs. In contrast, AdGFP-treated mice showed smaller and less defined granulomas. However, when compared to the total amount of granulomas, there were no differences between groups (Figure 3B).

### 3.3. Immune Regulation by AdTNF Treatment in a Murine Model of Progressive MDR-TB

**Figure 4:**
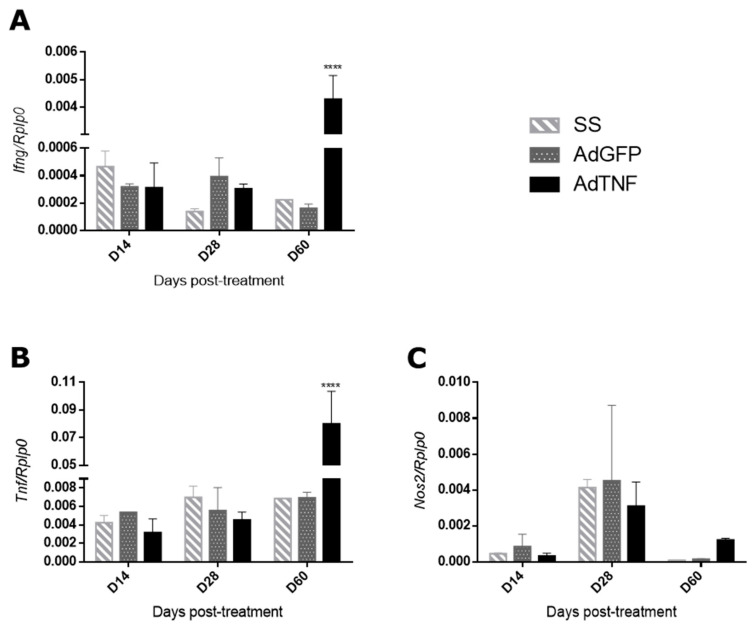
Gene expression of pro-inflammatory cytokines in the different experimental groups. Gene expression quantification by qPCR using the 2^^(−ΔCt)^ method relative to *Rplp0* (housekeeping gene) of (**A**) *Ifng*, (**B**) *Tnf* and (**C**) *Nos2* at day 14, 28 and 60 of treatment with AdTNF, AdGFP or SS. At day 60 of treatment, a significant increase in gene expression was induced by AdTNF treatment. Asterisks represent statistical significance (**** *p* < 0.0001, two-way ANOVA).

TNF is a pleiotropic cytokine that participates in several inflammatory processes, including bacterial infections, such as TB [4]. Thus, the administration of AdTNF is expected to increase the immune protective responses favoring Mtb containment and elimination. In order to assess this, gene expression in the lung of IFN-γ, iNOS and TNF was analyzed by qPCR. IFN-γ and TNF expression were higher at day 60 post-treatment in the AdTNF group when compared to AdGFP (Figure 4A,B); for iNOS expression, there were no differences at any time point (Figure 4C).

**Figure 5:**
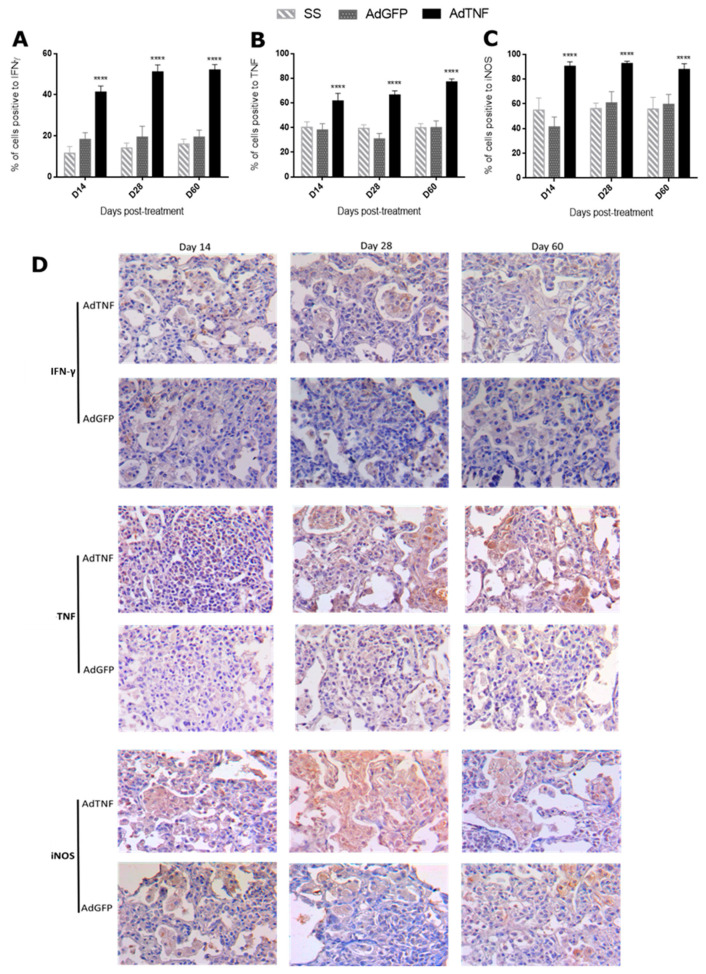
Percentage of immunostained cells to pro-inflammatory cytokines and iNOS in pneumonic areas of the different experimental groups. Percentage of immunostained cells to (**A**) IFN-γ, (**B**) TNF and (**C**) iNOS in the pneumonic areas of mice infected with MDR-TB after the indicated days of treatment with one dose of AdTNF, AdGFP or SS administered by intratracheal route. AdTNF induced a significant increment of immunostained cells in all the evaluated time points. Asterisks represent statistical significance (**** *p* < 0.0001, two-way ANOVA). (**D**) Representative microphags (40×) of immunostaining detection (brown cytoplasm peroxidase staining) of the indicated cytokine and iNOS in pneunmonic areas comparing AdTNF and AdGFP.

Considering these results, we decided to look for the protein by immunohistochemistry and stained cell counting in areas of pneumonia. In Figure 5A is shown the percentage of cells expressing IFN-γ that were higher in the AdTNF group at day 7, 28 and 60 post-treatment (2.3, 2.6 and 2.7 times higher, respectively) when compared to the AdGFP group; a similar response was observed in TNF-expressing cells (Figure 5B), being 1.6, 2.1 and 1.9 times higher at each time point for mice treated with AdTNF. Finally, for iNOS (Figure 5C), the percentages of cells expressing this protein in the AdTNF group were 2.2 times higher at day 7 and 1.5 times higher at days 28 and 60 post-treatment. Representative images of areas of pneumonia of cells expressing IFN-γ in Figure 5D are mainly macrophages and are more notorious in AdTNF treatment. TNF-expressing cells in mice treated with AdTNF are mostly macrophages along with epithelial cells, as expected from gene therapy; however, lymphocytes, which are not target cells of adenoviral vectors, were also positive for TNF expression, indicating a better response from mice treated with this vector, in contrast to the AdGFP vector, which did not show the same pattern of cell expression and most macrophages were negative for TNF expression. Similarly, iNOS-expressing cells in areas of pneumonia (Figure 5D) are macrophages; AdTNF therapy induced that almost all macrophages expressed iNOS, which is a key enzyme in the elimination of mycobacteria by the production of nitric oxide reactive species.

Differences between gene expression in total lung and cell expression of protein in areas of pneumonia indicated that AdTNF treatment favors Mtb containment and elimination, while AdGFP (control group) has an uncontrolled response that involves more lung damage and inefficient granuloma formation, as seen in Figure 2C and Figure 3A. Responses in cytokine expression during Mtb infection are different from healthy mice (Supplementary Figure S1), which have a low basal expression of *Tnf* and *Nos2*, and *Ifng* could not be detected by qPCR; TNF, IFN-γ and iNOS protein production in healthy mice is mainly by interstitial and alveolar macrophages, as well as lymphocytes.

### 3.4. The Effect of AdTNF and Second-Line Antibiotics Administration in a Murine Model of Progressive MDR-TB

**Figure 6:**
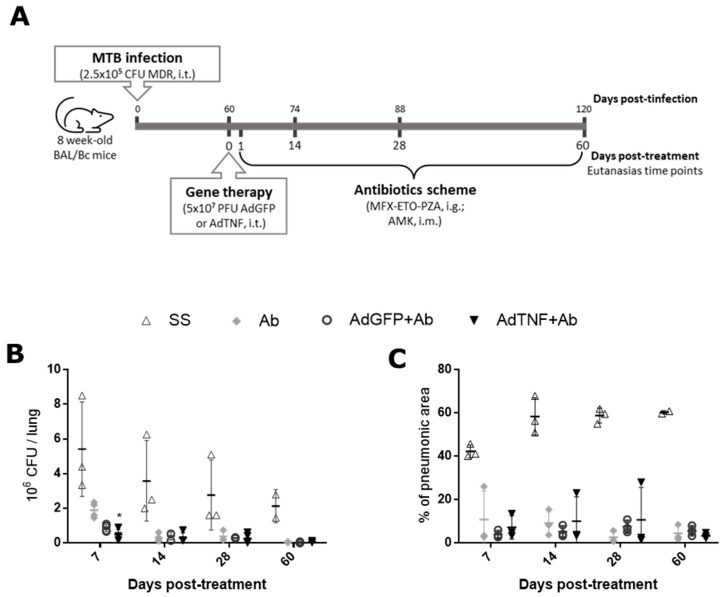
Effect of combined therapy of antibiotic and AdTNF treatment. (**A**) 8-week-old BALB/c mice were infected intratracheally (i.t.) with 2.5 × 10^5^ CFU of MDR mycobacteria, considering 6 mice per time of euthanasia. A single dose of gene therapy (AdTNF or AdGFP) was administered at day 60 post-infection, plus an antibiotic scheme by intragastric route of moxifloxacin (MFX), ethionamide (ETO), pyrazinamide (PZA) and amikacin (AMK) through intramuscular route, administered daily. Results shown are a representative kinetics from 2 independent experimental repetitions. (**B**) Bacillary loads determined by CFU counts in the indicated time points after treatment. (**C**) Extension of tissue damage represented by the percentage of lung surface affected by pneumonia. Asterisks represent statistical significance (* *p* < 0.05, two-way ANOVA).

Gene therapy with TNF showed a better disease course; however, mice did not eliminate Mtb infection. Thus, we performed a combined therapy of adenoviral vectors and 2nd-line antibiotics. BALB/c mice were infected with 2.5 × 10^5^ bacteria of MDR-Mtb, then at day 60 post-infection, mice were treated with a single dose of 5 × 10^7^ PFU/mice of AdTNF or AdGFP, or saline solution intratracheally, along with a daily dose of an intragastric antibiotic mix of moxifloxacin, ethionamide, pyrazinamide and amikacin through intramuscular route (Figure 6A).

As expected, antibiotic treatment reduced bacterial load considerably during all times of treatment when compared to the saline solution group (Figure 6B). CFU count diminished faster at day 7, by 1.8 times, when mice were treated with AdTNF plus 2nd-line antibiotics (AdTNF+Ab) than when mice were treated with AdGFP plus antibiotics (AdGFP+Ab); at days 14, 28 and 60, there were no differences between the two groups. The percentage of pneumonia was reduced in all groups that received 2nd-line antibiotics plus any other gene therapy, but there were no differences between AdGFP+Ab and AdTNF+Ab (Figure 6C). This indicates a synergic effect in shorter times of treatment (day 7), that could result in shorter times of treatment.

## 4. Discussion

MDR-Mtb is resistant to at least two of the first-line antibiotics (rifampicin and isoniazid); this is a growing health problem, since in 2021, there were 450,000 new cases, but it can also lead to the development of extensively drug-resistant strains (XDR-TB) that are resistant to second-line antibiotics like fluoroquinolones and aminoglycosides. Strategies for faster and more secure treatments are of great importance, since current treatments involve the use of two or more antibiotics (rifampicin, isoniazid or pyrazinamide) for large periods of time (6 months to even 12 months, depending on the severity of the disease); however, when an MDR-TB strain is diagnosed, treatment involves the use of at least three of the 2nd-line antibiotics (ethionamide, amikacin, moxifloxacin, levofloxacin, ethambutol, among other fluoroquinolones or aminoglycosides) for a period of 18 months approximately, depending on the Mtb strain and severity of the disease; this very long time of treatment has several consequences, such as high cost and toxicity, turning into a cycle when a patient abandons treatment due to toxicity or any other inconformity, and probabilities of a new drug resistance emerging become larger [1,20].

Gene therapy is a novel treatment solution for several types of diseases like cancer, infections, genetic disorders or vaccination [21,22,23]. Viral vectors are a tool that has become a safer and more precise option for the delivery of genes of interest; in our study, we used an adenoviral vector Ad5 serotype of the second generation that has deleted the E1 and E3 early-genes; this modification turns it into a non-replicative virus that also codifies for the flag gene GFP (green fluorescent protein), under the cytomegalovirus promoter, which is of high-level expression [24]. Adenoviral viruses have a natural tropism to epithelial cells and alveolar macrophages in the respiratory airway; this characteristic makes them a great system for gene therapy for TB pulmonary infection [25].

Here, we show that adenoviral vector codifying for TNF has a protective effect during pulmonary TB in a murine model of infection with MDR-Mtb, by up-regulating cells expressing a key protective cytokine like IFN-γ in pneumonic areas (Figure 5A). IFN-γ is a pro-inflammatory cytokine produced by CD4+ Th1 cells, CD8+ T cells, NK cells, B cells and antigen-presenting cells (APC) [26]; it has several anti-microbial functions like increasing production of reactive oxygen species and reactive nitrogen species by macrophages, along with strengthening antigen presentation to T cells [27]. Mtb is an intracellular pathogen and IFN-γ is also a significant factor for Mtb elimination [28]. In humans, severe TB correlates with an increase in IFN-γ in plasma and bronchoalveolar fluid [29,30]; without antibiotic treatment, disease severity progresses, and peripheral blood mononuclear cells become unresponsive and decrease the production of this cytokine [31,32]. This could explain the differences observed between gene expression in the whole lung by qPCR and protein expression by cells in pneumonic areas (Figure 4A and Figure 5A), indicating that AdTNF therapy prompts an adequate response in the site of infection, reducing bacterial load and uncontrolled expression of pro-inflammatory cytokines, preventing cell exhaustion and chronic disease outcomes like those observed in AdGFP or SS mice.

In accordance with IFN-γ observations, cells expressing the inducible nitric oxide synthase (iNOS) were also elevated in areas of pneumonia in mice treated with AdTNF (Figure 5C). The production of this enzyme is induced by pro-inflammatory cytokines or by the recognition of certain pathogen-associated molecular patterns, like lipopolysaccharide (LPS) from bacteria [33]. It is expressed by T cells, macrophages and dendritic cells; when macrophages express iNOS, a high amount of nitric oxide (NO) is produced, which participates in microbial elimination during phagocytosis by reacting with superoxide to form reactive nitric species (ONOO^−^) that can produce DNA and protein damage [34,35]. During Mtb infection, macrophage expression of iNOS is crucial for bacteria elimination [36]. Our results showed the induction of this enzyme in cells of mice treated with AdTNF, particularly in macrophages located in areas of pneumonia, which is an efficient response for the control and elimination of Mtb. This response was not observed when gene expression of iNOS was determined using total lung homogenates, but as seen in Figure 2D, the percentage of lung surface affected by pneumonia was lower in the AdTNF group, along with a lower bacterial load (Figure 2C), so inflammatory consolidation in the lungs of AdTNF mice is lower and pro-inflammatory molecules are more highly expressed in these areas where bacteria are more numerous.

TNF is a pro-inflammatory cytokine recognized for its key role in TB granuloma formation [37]. The relevance of this cytokine during Mtb progressive infection or even in latent infection has been shown during treatment of autoimmune disease where anti-TNF therapy is applied, and there is a higher incidence of TB reactivation [3,38]; in mice deficient of TNF receptor (TNFRp55^-/-^), there was also deficient granuloma formation with a lethal outcome [39]. It is known that TNF activates NF-kB and AP-1 transcription factors and induces apoptosis or necrosis, depending on the molecular microenvironment [40,41]. All these functions of TNF are of high relevance during Mtb early, progressive and chronic infection; any misfunction could lead to a more severe course presenting more tissue damage in the lung, or bacteria dissemination [42]. Due to all these properties of TNF, we chose it as a gene of interest for gene therapy delivery in our model of murine progressive TB using an MDR clinical isolate. As mentioned before, AdTNF therapy produced total animals’ survival, lower bacterial load and lesser pneumonia. Interestingly, granuloma formation was also better in the AdTNF group, being bigger than the control groups (AdGFP and SS), indicating a better conformation that efficiently contains and eliminates Mtb, even though the numbers of granulomas were not different.

Elevated TNF production during TB infection has been linked in some studies with the induction of necrotic areas in the lung; in a zebrafish model infected with *M. marinum*, the injection of extra TNF protein induced the production of mitochondrial reactive oxygen species (mROS) that were involved in macrophage necrosis induction [43]. This scenario was a possibility; however, we did not find any signs of necrotic events in the lungs of mice infected and treated with AdTNF when histologic analysis was performed; it is important to consider the dose of viral vector administered in order to prevent the overexpression of TNF.

As mentioned before, TB treatment involves several antibiotics for a long period of time; this can lead to secondary effects like gastrointestinal problems, exanthema, severe hepatotoxicity or nerve inflammation [2,44]. Novel therapies to improve TB treatment are necessary; in this context, combined therapy of antibiotics and adenoviral vectors (gene therapy) has been proved before to be efficient in our laboratory for other cytokines like IFN-γ [15], osteopontin [16] and GM-CSF [45], and for antimicrobial peptides like cathelicidin (LL37) and beta defensin-3 [46], confirming gene therapy as a tool for a faster improvement in experimental progressive TB. Since AdTNF therapy did not totally cure the disease, we tested the combined therapy with second-line antibiotics (moxifloxacin, ethionamide, pyrazinamide and amikacin), administered daily in combination with a single dose of 5 × 10^7^ PFU/mice of AdTNF at day 60 post-infection with MDR-Mtb. At day 7 post-treatment, AdTNF+Ab mice had lesser bacterial load than mice treated with the control vector (AdGFP+Ab) and a bigger difference was seen in comparison with mice that only received antibiotic treatment. However, antibiotic treatment cleared bacterial load very efficiently, so differences in tissue damage such as percentage of lung area affected by pneumonia were not observed (Figure 6B).

Mouse models are a practical and efficient tool that have helped scientific research for many years; however, a significant limitation of our study is that the results are not always directly an indicator of what would happen in humans due to species differences, conditions, nutrition and other lifestyle situations, so gene therapy has to be used cautious and preferably with an intermediary step in non-human primate models. Another limitation is that we used first-generation recombinant adenoviruses and currently there are safer and cleaner systems that can be used for future and complementary studies, for instance, without the GFP gene expression, or with more genes deleted or even the lack of the adenoviral genome with the advantage of been less immunogenic.

In conclusion, our results showed that gene therapy based in recombinant AdTNF, reactivates protective immune responses by increasing IFN-γ and iNOS expression in inflammatory cells located in pneumonic areas, which leads to a better formation of granulomas that efficiently contain and eliminate Mtb, resulting in lesser bacterial load and lung damage (percentage of pneumonia) that leads to total survival of mice. These results sum up the previous results of gene therapy against TB, while considering in the future a mixed therapy of several genes of interest that could regulate and potentiate the immune responses for faster control and elimination of Mtb, and also be efficient against MDR-TB strains that can reduce the time or dosages of antibiotics, diminishing the probabilities of abandonment and toxicity along with the surge prevention of MDR or XDR mycobacteria.

## Data Availability

Data are contain within the article and supplementary materials.

## Supplementary Materials

The following supporting information can be downloaded at: https://www.mdpi.com/article/10.3390/microorganisms11122934/s1, Figure S1: Cytokine expression of 8-week-old non-infected BALB/c mice.
