# Supplementary material for: Adenoviral Vector Codifying for TNF as a Co-Adjuvant Therapy against Multi-Drug-Resistant Tuberculosis
Source: Microorganisms. 2023 Dec 7;11(12):2934. doi: 10.3390/microorganisms11122934 (PMC10745769; doi:10.3390/microorganisms11122934)
Supplement: Supplementary file 1 [file microorganisms-11-02934-s001.zip › microorganisms-2717034-supplementary.pdf]

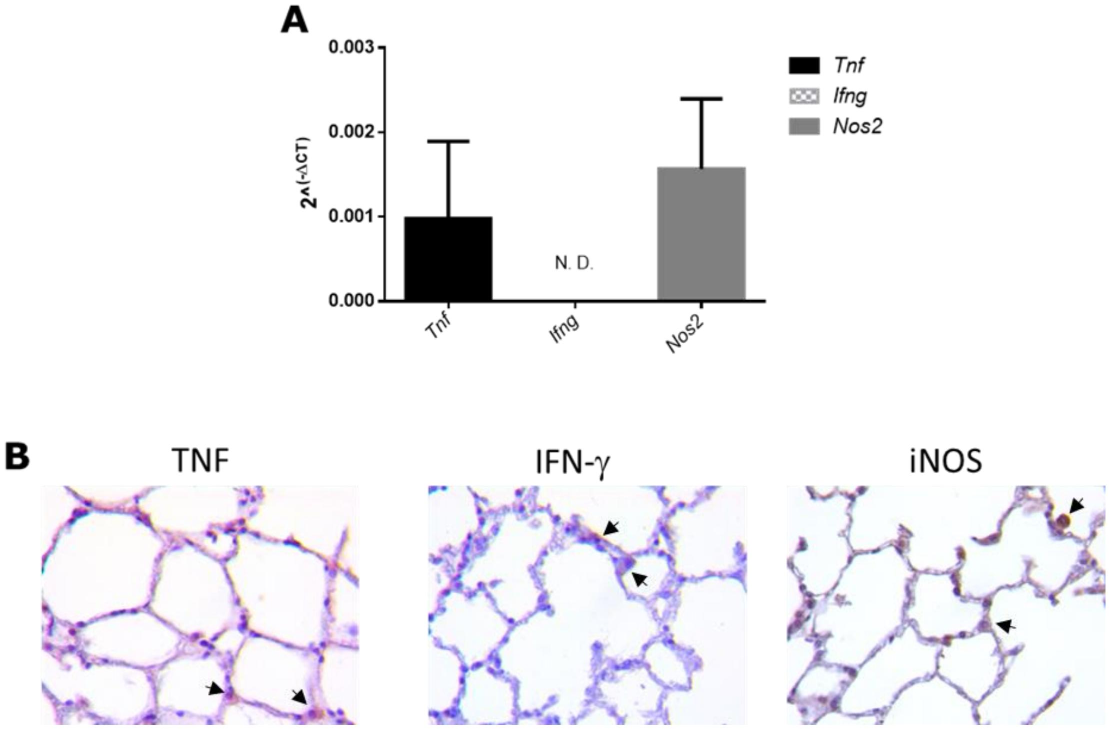

Supplementary figure S1. Cytokine expression of 8-week-old non-infected BALB/c mice. **(A)** Gene expression of *Tnf*, *Ifng* and *Nos2* in lung of non-infected mice by qPCR with the $2^{(\Delta CT)}$ method relative to the endogenous gene *Rplp0*. **(B)** Representative image of immunohistochemistry in lung of non-infected mice, showing TNF, IFN-γ and iNOS expression by interstitial and alveolar macrophages (black arrow).
